# How to measure vessel flow with CMR phase contrast imaging

**DOI:** 10.1186/1532-429X-17-S1-T5

**Published:** 2015-02-03

**Authors:** Chris B Lawton, Nathan E Manghat, Chiara Bucciarelli-Ducci, Mark Hamilton

**Affiliations:** 1 CARDIAC MRI UNIT, Brstol Heart Institute, Bristol, UK

## Background

Accurately measuring blood flow with CMR is paramount in patients with valvular disease. These flow measurements are important components of a CMR report and provide the referring clinicians with valuable information in which to base further clinical management. This poster presents a guide on how to measure blood flow accurately based on our experience at the Bristol Heart Institute.

## Methods

### Slice positioning

**Figure 1:**
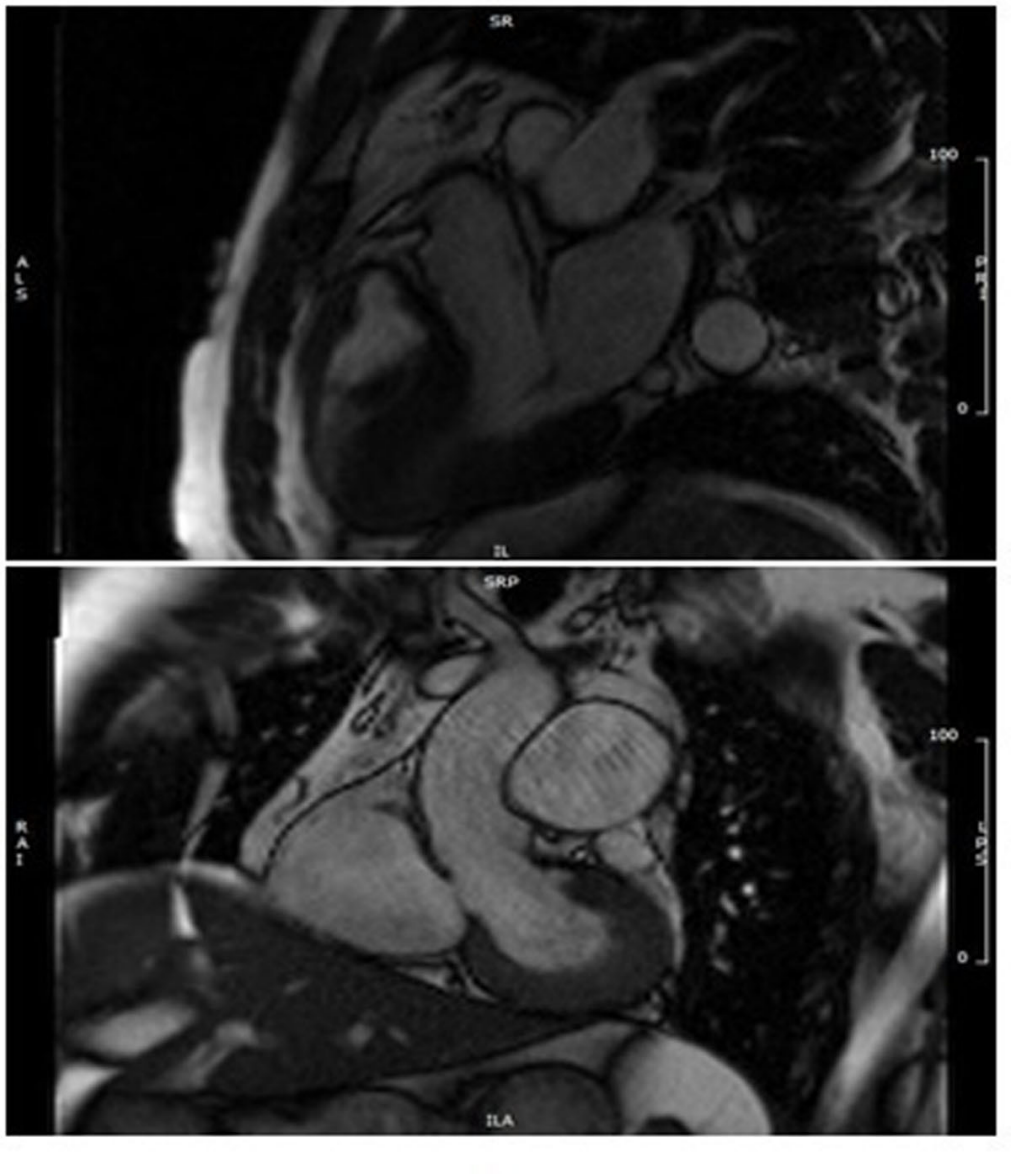
Position slice on two orthogonal planes.

**Figure 2:**
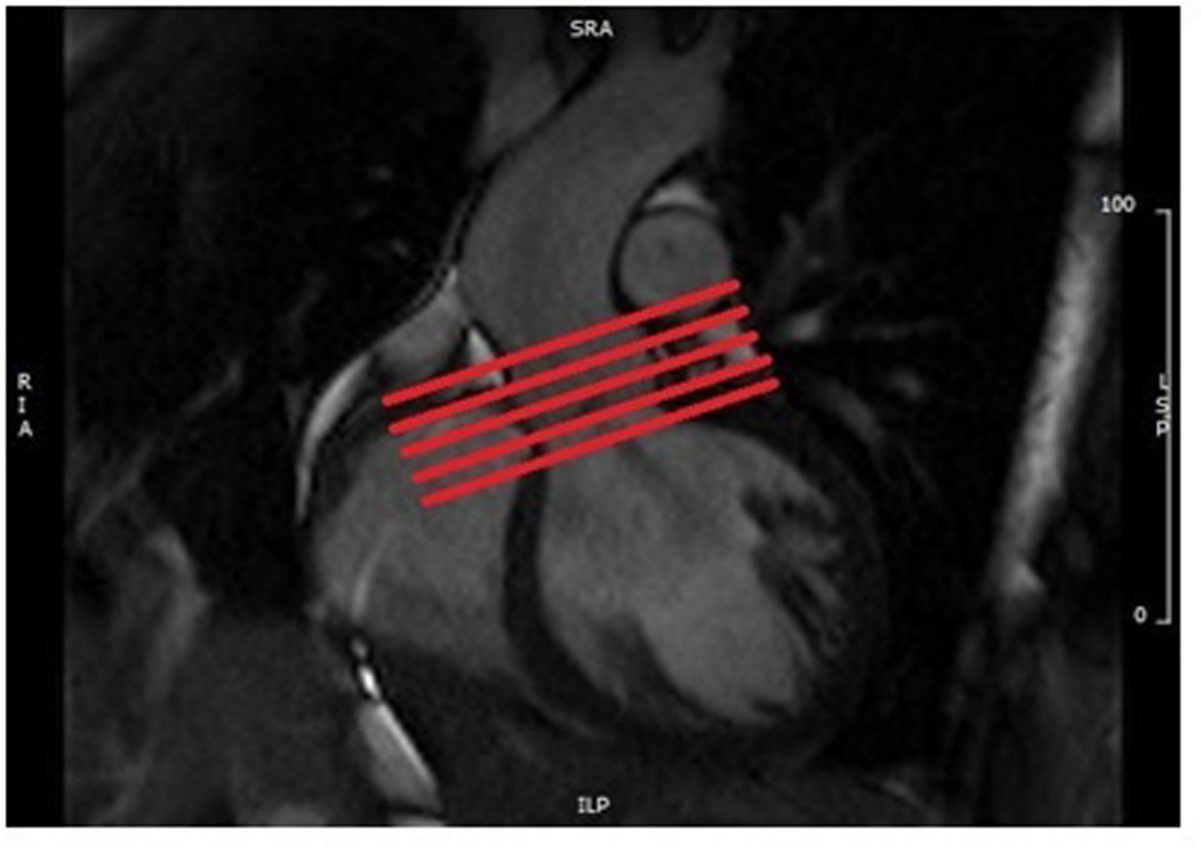
Positioning of a stack of breath held flows across the AV.

It is important that the phase contrast slice is perpendicular to the vessel and therefore this should be checked on two orthogonal cine images [Fig 1]. Oblique deviation of the slice from the orthogonal plane of the vessel can cause up to a 10% error in the peak flow and flow rate measurements [Lotz 2002]. To measure peak velocity the slice must be positioned at the tips of the leaflets in systole. For this reason a breath held flow sequence is required planned from the breath held cines. Free breathing is inappropriate due to through plane motion. To measure volume of flow the slice must be positioned in such a place where the flow is laminar to reduce the effects of turbulent chaotic flow leading to loss of signal and loss of data. In many diseased valves with turbulent stenotic and regurgitant jets no single slice position can estimate both antegrade and retrograde flow accurately. Thus in this situation a stack of breath held flows is prescribed from proximal to distal to the valve. [Fig 2]. Locating the valve annulus in systole on cine images also provides a valuable location for consistent slice positioning.

### Velocity Encoding (Venc.) matching

The velocity encoding (Venc) range should be chosen to match the peak velocity as closely as possible. If the Venc is too high the image will become noisy with high background error, too low, and an artefact known as aliasing will corrupt the measurement flow data, rendering it un-diagnostic. To ensure the Venc is matched correctly, run a flow scout sequences at 4 different Venc settings [Fig 3]. Alternatively start with a low Venc, if there is aliasing repeat in steps of 50 until it disappears.

### Free Breathing & Breath hold flows

Free breathing flow offers higher temporal and spatial resolution than a breath hold flow as a greater number of heart beats are used to acquire the data. As it is also measured over multiple respiratory and cardiac cycles it would appear to be the method of choice to measure arterial valve function. However when there is turbulent flow or when peak velocity estimation is required it can produce misleading data. In this situation a stack of breath hold flows across the valve can be employed. This optimises slice position for peak velocity and peak flow measurement.

## Results

No results as this is a reference poster.

## Conclusions

Accurate positioning of the slice and selecting the correct velocity encoding are paramount steps when measuring blood flow. Due care and diligence needs to be shown to achieve accurate results.

## Funding

National Institute for Health Research, Biomedical Research Unit.

